# Editorial: Novel rehabilitation approaches for non-communicable diseases in the era of precision medicine

**DOI:** 10.3389/fmed.2026.1796605

**Published:** 2026-02-09

**Authors:** Júlio Belo Fernandes, Tiago F. Outeiro, Cristina Lavareda Baixinho, Catarina Godinho

**Affiliations:** 1 Egas Moniz Center for Interdisciplinary Research (CiiEM), Almada, Portugal; 2 Egas Moniz School of Health and Science, Almada, Portugal; 3 Nurs^*^ Lab, Almada, Portugal; 4 Faculdade de Medicina e Ciências Biométicas, Algarve Biomedical Center Research Institute (ABC-Ri), Algarve Biomedical Center (ABC), University of Algarve, Faro, Portugal; 5 Department of Experimental Neurodegeneration, Center for Biostructural Imaging of Neurodegeneration, University Medical Center Göttingen, Göttingen, Germany; 6 Faculty of Medical Sciences, Translational and Clinical Research Institute, Newcastle University, Newcastle Upon Tyne, United Kingdom; 7 Deutsches Zentrum für Neurodegenerative Erkrankungen (DZNE), Göttingen, Germany; 8 Nursing Research, Innovation and Development Centre of Lisbon (CIDNUR), School of Nursing, University of Lisbon, Lisbon, Portugal

**Keywords:** adaptive interventions, artificial intelligence (AI), digital health (eHealth), functional recovery, non-communicable diseases (NCD), precision medicine and genomics, precision rehabilitation, rehabilitation

## Introduction

Non-communicable diseases remain the dominant cause of long-term disability and health-service utilization worldwide (1). Their trajectories are shaped by multimorbidity, fluctuating symptoms, and progressive functional limitations—features that challenge rehabilitation models built on standardized protocols and averaged responses (2). While rehabilitation has long been recognized as a core component for the management of non-communicable diseases (3), its operationalization has often lagged behind advances in disease stratification and outcome prediction (4, 5). In contrast, precision medicine has promoted the integration of biological, phenotypic, behavioral, and contextual information to improve risk stratification, predict outcomes, and personalize care (6, 7). However, translating these principles into rehabilitation is not simply a matter of adding biomarkers or technology. It requires aligning intervention selection, intensity, timing, and delivery with the person's evolving capacity, goals, and living context, in an active cooperation to maximize the outcomes of the intervention.

The Research Topic, *Novel Rehabilitation Approaches for Non-Communicable Diseases in the Era of Precision Medicine*, brings together 14 contributions, including work by Anghelescu et al., Bertuccio et al., Chen et al., Dan et al., Fernandes et al., Guo et al., Huang et al., Li et al., Micheluzzi et al., Yepez et al., Zhang et al., Zhao et al., Zhencheng and Aiguo, and Zhu et al., suggesting that precision in rehabilitation should be understood not as a replacement of existing paradigms, but as an expansion of the operational space in which precision medicine principles are enacted, tested, and adapted over time.

Across the contributions, precision appears less as a discrete technology and more as a guiding logic for aligning interventions with individual variability. In this context, precision rehabilitation can be understood as a dynamic, person-centered process that translates precision medicine principles into rehabilitation practice. It involves the ongoing integration of biological risk profiles, functional assessment, behavioral and contextual determinants, and lived experience to support adaptive decision-making, intervention tailoring, and meaningful participation across rehabilitation trajectories.

In musculoskeletal rehabilitation, for example, the focus shifts from generic endorsements of exercise toward comparative and stratified approaches that consider symptom profiles, functional priorities, feasibility, and delivery context. Rather than asking whether exercise is effective, the underlying question becomes which modality, delivered in which format, is most appropriate for a given person at a given point in time. This reframing reflects a broader movement away from protocol-centered care toward adaptive intervention design.

Predictive modeling further illustrates this operational expansion of precision. The increasing use of machine learning and composite biomarkers highlights how routinely collected data can be leveraged to identify individuals at higher risk of pain, functional decline, or adverse outcomes. From a rehabilitation perspective, the value of such models lies not only in prediction but also in their potential to inform the timing, intensity, and prioritization of interventions. Precision, in this sense, is not an endpoint but a decision-support process that must remain interpretable, clinically meaningful, and responsive to change.

An additional dimension that emerges across these contributions is temporality. Precision rehabilitation is inherently dynamic, as individuals with non-communicable diseases move through phases of stability, exacerbation, recovery, and adaptation. Interventions that are appropriate at one point in the disease or rehabilitation trajectory may become ineffective or burdensome at another. This highlights the need for longitudinal assessment frameworks and adaptive intervention models that can evolve alongside functional status, goals, and contextual constraints. In this sense, precision is not a static classification, but a continuous process of recalibration over time.

Cardiopulmonary rehabilitation provides an obvious illustration of how precision extends beyond biology. Outcomes in these pathways are strongly influenced by adherence, health literacy, social support, and symptom self-monitoring, factors that are rarely captured by biological markers alone. Evidence from the studies by Dan et al. and Huang et al. underscores the role of behavioral and contextual determinants in shaping functional recovery, reinforcing the need for precision rehabilitation to integrate social and cognitive dimensions alongside physiological data. Digital platforms and remote delivery models further expand this operational space by enabling tailored support, co-creation, and sustained engagement, especially for populations facing access barriers.

Neurorehabilitation contributions from the studies of Anghelescu et al. and Fernandes et al. highlight another critical dimension: assessment. Precision rehabilitation is as much an assessment challenge as an intervention challenge. Without sensitive, multidimensional, and responsive measures, personalisation risks becoming speculative. Advances in artificial intelligence–supported assessment and biomarker-oriented frameworks illustrate how greater measurement granularity can support adaptive decision-making across disease trajectories. These developments point toward a future in which assessment is not a static gateway to intervention, but a continuous feedback mechanism guiding rehabilitation over time.

These shifts also have important implications for the role of rehabilitation professionals. Precision-oriented approaches reposition clinicians not as implementers of fixed protocols, but as integrators of biological, functional, behavioral, and experiential information across time. Clinical judgement, shared decision-making, and the ongoing negotiation of goals become central competencies in precision rehabilitation, particularly in the context of multimorbidity and long-term conditions. Supporting this role will require education pathways, organizational cultures, and service models that value adaptive reasoning alongside technical expertise.

Mechanistic perspectives emerging from contributions by Zhencheng and Aiguo and Zhang et al. further extend the precision agenda by linking molecular signaling and systemic inflammatory processes to recovery trajectories, symptom modulation, and longer-term outcomes. While Zhencheng and Aiguo provide a primarily conceptual synthesis, Zhang et al. offer population-level evidence using composite inflammation indices as mechanistically informed proxies for risk and prognosis. Together, these articles help bridge basic science and rehabilitation practice and underscore the need for future research that connects mechanistic biomarkers with clinical phenotypes and differential responsiveness to intervention. Crucially, they emphasize that biological precision is not sufficient unless translated into actionable rehabilitation strategies.

The contributions of Fernandes et al. and Zhao et al. also foreground conditions such as dysphagia, where heterogeneity, neurological complexity, and feasibility constraints demand highly targeted approaches. Pilot trials and detailed case-based reasoning illustrate how mechanism-informed, person-centered interventions can be developed and tested in complex clinical contexts. These studies demonstrate the value of feasibility-driven designs, careful phenotyping, and safety monitoring as prerequisites for scaling precision-oriented interventions.

Beyond the biological and functional domains, experiential dimensions of rehabilitation are receiving increasing attention. Qualitative insights into immersive and technology-assisted rehabilitation environments highlight how embodiment, motivation, emotional engagement, and meaning-making shape participation and adherence. From this perspective, precision rehabilitation encompasses experiential alignment: adapting interventions not only to measurable characteristics, but also to how individuals perceive, tolerate, and emotionally respond to therapeutic demands. This dimension is particularly relevant as digital and immersive technologies become more prevalent in rehabilitation practice.

At the population level, large-scale analyses linking inflammatory markers to long-term mortality among people with coexisting non-communicable diseases further demonstrate how precision approaches can inform prognostic profiling. For rehabilitation, such insights may support more nuanced decisions regarding intervention intensity, monitoring, and long-term follow-up, particularly in the context of multimorbidity. However, these advances also raise important questions regarding equity, interpretability, and implementation across diverse health systems.

As precision-oriented rehabilitation approaches mature, challenges of implementation and equity become increasingly salient. Data-intensive models, digital delivery platforms, and advanced assessment tools risk exacerbating existing disparities if access, literacy, and contextual variability are not explicitly addressed. Ensuring that precision rehabilitation contributes to more equitable outcomes will require deliberate attention to implementation science, co-design with end users, and alignment with the realities of diverse health systems.

Collectively, the contributions to the Research Topic suggest that precision rehabilitation should be understood as an integrative and adaptive process rather than a fixed model. Rather than supplanting precision medicine, rehabilitation expands its operational domain by translating predictive and stratification insights into dynamic, person-aligned action. This expansion requires moving beyond single outcomes and isolated innovations toward multidimensional assessment, adaptive dosing over time, meaningful engagement of individuals, and equity-aware implementation.

Looking ahead, progress in precision rehabilitation will depend not only on technological novelty but, equally, on integration and patient-engagement. Future research should prioritize frameworks that connect biological, functional, behavioral, contextual, and experiential data. It should also develop assessment systems capable of guiding real-time adaptation, and test interventions within the complexity of real-world practice. By doing so, rehabilitation can fulfill its role as a critical translational space—where precision principles are not only predicted, but lived, negotiated, and refined in partnership with people living with non-communicable diseases.
